# Docosahexaenoic Acid in Formulas for Term Infants: The Way from Pioneer Idea to Mandatory Dietary Recommendation

**DOI:** 10.3390/life13061326

**Published:** 2023-06-05

**Authors:** Tamás Decsi, Tamás Marosvölgyi, Éva Szabó

**Affiliations:** 1 Department of Pediatrics, Medical School and Clinical Centre, University of Pécs, 7623 Pécs, Hungary; 2 Cochrane Hungary, Clinical Centre, University of Pécs, 7623 Pécs, Hungary; 3 Institute of Bioanalysis, Medical School, University of Pécs, 7624 Pécs, Hungary; marosvolgyi.tamas@pte.hu; 4 Department of Biochemistry and Medical Chemistry, Medical School, University of Pécs, 7624 Pécs, Hungary

**Keywords:** arachidonic acid, cognitive development, docosahexaenoic acid, infant nutrition, long-chain polyunsaturated fatty acids, neurodevelopment, visual development

## Abstract

Docosahexaenoic acid (DHA) is a novel mandatory constituent of breast-milk-substitute infant formula in Europe. The aim of the present narrative review was to summarize available data in connection with the background of the novel European mandatory dietary recommendation to add at least 20 mg/100 kcal (4.8 mg/100 kJ) DHA to infant formula. The literature search with the expression “docosahexaenoic acid with (infant or human milk or formula)” revealed nearly 2000 papers, including more than 400 randomized controlled trials (RCTs). DHA is a persistent constituent of human milk (HM) with a worldwide mean level of 0.37% (standard deviation: 0.11%) of all fatty acids in HM. RCTs on supplementing DHA to lactating women showed some indications, though no direct evidence of the beneficial effect of enhanced HM DHA on the development of breastfed infants. The most-recent Cochrane review of RCTs investigating the effect of DHA supplementation to infant formula for full-term infants reported no evidence for recommending supplementation. The controversy between the Cochrane view and the actual recommendation may be related to the numerous hurdles in organizing high-quality studies in this field. On the basis of the official food composition recommendation, today in Europe, DHA should be considered as a fatty acid essential for infants.

## 1. Docosahexaenoic Acid: Novel Mandatory Constituent of Infant Formula in Europe

Docosahexaenoic acid (C22:6n-3, DHA) is the bioconversion product of complex biosynthetic mechanisms from the initial essential fatty acid (EFA), alpha-linolenic acid (C18:3n-3, ALA) [1]. While there is an ongoing discussion about whether ALA interconversion might be sufficient on its own to cover n-3 long-chain polyunsaturated fatty acid (LCPUFA) needs in humans [2], the utmost majority of n-3 LCPUFA supplementation trials in pregnancy, lactation, and infancy included preformed DHA [3]. Today, there are various DHA delivery systems applied to improve the bioavailability of DHA and support the role of its functionality [4].

Probably the most-widely discussed recommendation of DHA supplementation on healthy diets has already been regulated by the European Union (EU), and these new mandates went into effect on 22 February 2021. This regulatory measure means that all infant formula and follow-on formula that are available and could be purchased in the EU must contain at least 20 mg/100 kcal (4.8 mg/100 kJ) and at most 50 mg/100 kcal (128 mg/100 kJ) DHA [5]. With this decision, DHA became a nutritional constituent of infant formula that is a mandatory requirement similar to vitamins, trace elements, essential amino acids, and EFAs. In the case of DHA for full-term infants, it took about 30 years to go from the first suggestion of supplementation to dietary recommendation (vide infra). A somewhat shorter timespan between the idea and action might have benefited some formula-fed infants who failed to receive preformed DHA during a critical period of the maturation of visual and cognitive functions (vide infra).

While there are more than 30 already mandatory nutrition ingredients of infant formula [6,7], various other biologically active substances have also been associated with potential beneficial effects on infant growth [8] and health [9]. The characterization of the way from the probably first mentioning of DHA in connection with infant nutrition [10] to its mandatory inclusion into infant formula might provide ideas for further improvement of the nutritional composition of infant formula. In the present narrative review, we try to highlight such aspects of research on DHA in infant nutrition that might be utilized in connection with improving the nutrition of infants who are not breastfed.

## 2. Literature Search on Docosahexaenoic Acid in Relation to Infant Nutrition

**Figure 1:**
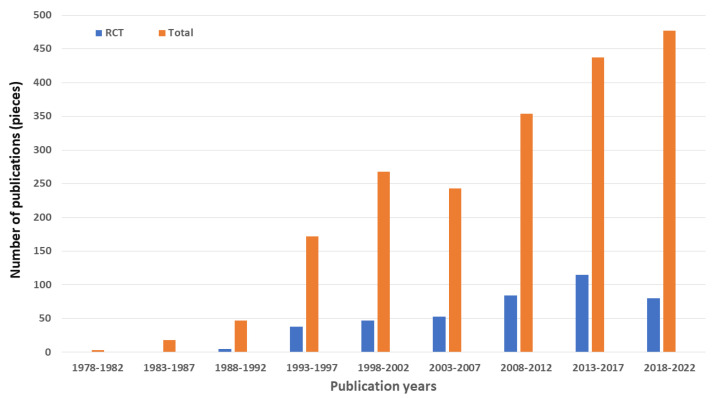
Number of randomized controlled trials (RCTs) and total number of papers in PubMed database on docosahexaenoic acid in connection with infant nutrition. The database was searched on 19 January 2023 by using the search expression “docosahexaenoic acid with (infant or human milk or formula)”.

The first mentioning of DHA dates back in PubMed and Embase to 1957 [11] (in SCOPUS to 1938 [12]). Today (19 January 2023), there are more than 18,000 papers in PubMed on DHA, including more than 1800 randomized controlled trials (RCTs). When the search is limited to papers with a potential connection to infant nutrition—search expression: “docosahexaenoic acid with (infant or human milk or formula)”—nearly 2000 papers including more than 400 randomized controlled trials (RCTs) can be identified (Figure 1).

The data shown in Figure 1 indicate very active clinical research on DHA in connection with infant nutrition. The absolute numbers of publications, i.e., about 40 to 80 publications including about 8 to 20 RCTs per year during the last 30 years, probably cannot tell much on their own about the research activities. It is to be noted, however, that, in the PubMed database, about 20% of the publications on DHA in connection with infant nutrition are RCTs, whereas the corresponding ratios in general DHA research are about 10%, in general infant nutrition research about 5%, and in the totality of the PubMed human database about 3%. Therefore, it can be concluded with good reason that the role of DHA in infant nutrition is not only actively discussed, but exceptionally actively investigated in clinical trials as well.

## 3. Docosahexaenoic Acid in Breastfed Infants

Human milk (HM) is also the compositional gold standard of infant formula; therefore, the consequent presence of a given ingredient in HM is a prerequisite of the inclusion of the same substance into infant formula. Moreover, any novel ingredient to be included into the nutritional composition of infant formula should be positively related either to infant growth and development or to the prevention of some pathological condition in breastfed infants.

### 3.1. Docosahexaenoic Acid in Human Milk

Data on the fatty acid (FA) composition of HM have been accumulating for more than 40 years [13]; today, extensive systematic reviews on HM DHA contents are available [14,15,16]. When data reported in 78 studies on 3746 women with lactation stages between 2 weeks and 18 months were reviewed together, worldwide mean levels of DHA within the FA composition of mature HM were found to be 0.37% (standard deviation (SD): 0.11%) [14]. Subgroup analysis of colostrum (1st to 6th days of lactation), transient HM (7th to 14th days of lactation), and mature HM (>14 days of lactation) in Chinese women participating in 35 studies showed mean DHA values as 0.62% (SD: 0.40%, *n* = 1079), 0.50% (SD: 0.21%, *n* = 627), and 0.47% (SD: 0.36%, *n* = 1085), respectively [15]. Only slightly different results were reported from a pooled data analysis from 55 studies worldwide; the weighted least-squares mean DHA contents were 0.51% (standard error of the mean (SEM): 0.04%, *n* = 943) in colostrum, 0.45% (SEM: 0.06%, *n* = 957) in transient HM, and 0.31% (SEM: 0.03%, *n* = 2397) in mature HM [16].

There are considerable geographical variabilities among DHA content in mature HM: among different regions of China, the mean values varied between 0.18% and 0.98% [15], whereas among different regions of the world, the lowest and highest mean least-squares HM DHA values were 0.1% and 0.98% [16]. Similar variability was found among European countries where the lowest and highest mature HM mean DHA values were 0.11% (Hungary) and 0.71% (Switzerland), respectively [16]. It is to be emphasized that HM DHA content is linearly correlated with dietary the DHA intakes of the lactating mothers [17].

### 3.2. Docosahexaenoic Acid Intake in the Breastfed Infant

Within the framework of a study carried out with well-defined methods in a given population, infantile DHA intakes can be calculated by measuring (a) the contribution of DHA to the FA composition of HM, (b) fat content in HM, and (c) HM intake of the infants. Within the framework of the European Childhood Obesity project, full-term infants who received at least 90% of all feedings in the first 3 months of life as HM were investigated [18], and the calculated DHA intakes were found to be very close to around 50 mg/day during the first 3 months of life (Table 1).

However, it is rather difficult for a number of reasons to generalize DHA intake data from the above-illustrated precise calculation to other populations. Firstly, there might be several-fold differences between populations in the contribution of DHA to the FA composition of HM (vide supra). Secondly, there might be considerable methodological differences in HM sampling, in the measurement of the milk volume consumed by the infant, and in determining the HM fat content (for detailed methodological considerations, see [19,20]). Thirdly, the at least 90% HM feeding during the first 3 months of life—i.e., the inclusion criterion set by Grote et al. [18] for their study—is rather the exception than the rule in breastfeeding practices today (vide infra).

Because cows’ milk (or soybean or rice) does not contain DHA, the traditionally manufactured infant formulas do not contain detectable amounts of DHA. Unless, the formula is specifically supplemented with preformed dietary DHA, the most uncertain factor influencing DHA intake in partially breastfed infants is the extent of the contribution of formula without preformed dietary DHA to their diet.

### 3.3. Effects of Docosahexaenoic Acid on the Breastfed Infant

N-3 LCPUFAs have been attributed a broad spectrum of important physiological roles including effects on cognitive functions [21], vascular endothelial processes [22], immune cell functions [23], and regulatory mechanisms within insulin-sensitive tissues [24]. A detailed discussion of these physiological effects of DHA within the human organism would extend far beyond the limits of the present review; the comprehensive references cited [21,22,23,24] are available as free full text in PubMed. Potential preventive, as well as therapeutic effects of n-3 LCPUFA supplementation have been advocated in connection with a long list of various pathological conditions and diseases, involving subjects and patients from the intrauterine to the elderly periods of human life [25].

The consequent presence of DHA in HM raises the question of its potential beneficial effect (or to put it in another way: evolutionary advantage) on the development of the infant; the multifaceted physiological role of DHA provides various mechanisms to explain such influence. Breastfeeding, at least in high-income settings, protects against otitis media, likely protects against type-2 diabetes mellitus, and overweight and obesity and likely improves the intelligent quotient by 2 to 3 percentage points [26]. At first glance, some of these beneficial effects might appear to be related to the presence of DHA in HM and the absence of it in traditionally manufactured infant formula. However, breastfed infants differ from those receiving formula not only in their nutrient intakes, but usually in several socio-economic factors influencing the young mother’s choice of the way of infant feeding. Therefore, a simple comparison of breastfed infants to those receiving formula cannot provide evidence on the potential effects of dietary DHA supply with HM. In contrast, controlled enhancement of the contribution of DHA to the FA composition of HM through increased dietary supply of DHA to lactating mothers may offer insight into the specific role of DHA in the development of breastfed infants [27].

In a Cochrane Database Systematic Review of eight RCTs involving 1567 women who received LCPUFA supplementation during lactation [28], long-term follow-up beyond 24 months did not reveal significant differences in language development, intelligence, problem-solving ability, psychomotor development, motor development, or in general movements. However, child attention scores were better at five years of age in the group of children whose mothers received about 200 mg/day DHA supplementation from delivery until 4 months postpartum (*n* = 60) than in the control group without DHA supplementation (*n* = 59) [29]. In the same study, the Bayley Psychomotor Development Index of 30-month-old children whose mothers received DHA were about 0.5 SD higher than that of children whose mothers received placebo [30].

Two further RCTs within the Cochrane review [28] failed to show significant differences between groups, but indicated some relationship of DHA to functional outcomes. When lactating mothers received DHA supplementation in five different doses (0, 0.2, 0.4, 0.9, and 1.3 g/day) between Day 5 and Week 12 postpartum, the Bayley Mental Developmental Index at 1 year was significantly and positively associated with 12 weeks’ breast milk DHA (r = 0.29, *p* = 0.04) and 12 weeks’ infantile erythrocyte DHA (r = 0.32, *p* = 0.02) [31]. When lactating women received either 800 mg DHA and 600 mg eicosapentaenoic acid (C20:5n-3, EPA) per day or placebo (olive oil) during the first 4 months of lactation, multiple regression analysis showed a highly significant positive association (*p* = 0.004) of infant visual acuity with infantile erythrocyte DHA at 4 months of age [32].

In summary, the data discussed above provide some indications, but no direct evidence of the potential beneficial effect of HM DHA on infantile development.

## 4. Docosahexaenoic Acid in Infants Fed Formula

The utmost medical priority in infant nutrition is to protect, support, and promote breastfeeding. Any effort invested into the modification of the composition of infant formula can be justified only if (a) there is an unavoidable need to use formula and (b) the modification can be positively related in infants fed formula either to infant growth and development or to the prevention of some untoward health outcome.

### 4.1. Contribution of Infant Formula to the Diet of Full-Term Infants

The World Health Organization (WHO) and the United Nations Children’s Fund recommended that children initiate breastfeeding within the first hour of birth and be exclusively breastfed for the first 6 months of life—meaning no other foods or liquids are provided, including water [33]. Optimal fulfilment of this recommendation would leave very little need for research on breast-milk-substitute infant formula. However, the current official public statement of the WHO indicates that only about 44% of infants aged 0 to 6 months are exclusively breastfed worldwide [34].

In Europe, a recent survey of 11 National Breastfeeding Committees and Representatives [35] indicated that only between 56% and 97% of infants in all countries received any HM, at the age of 4 months only 42% to 56%, whereas at the age of 6 months, only 13% to 39% of the infants were exclusively breastfed. The ratio of infants who did not receive any breastfeeding was between 19% and 42% at the age of 4 months and between 29% and 62% at the age of 6 months [35].

Breastfeeding indicators were also recently summarized by using all data sources (including, e.g., infant survey, breastfeeding survey, primary healthcare data, maternity hospital data) available from 51 out of the 82 high-income countries of the world [36]. The time period covered by the data points ranged from 1986 to 2019, and 71% of the countries had updated their indicators since 2015. Data for ever breastfeeding were reported for 46 countries with a median of 91%; among the European countries (*n* = 26), this value varied between 99% (Finland) and 60% (Northern Ireland and Republic of Ireland). Information for exclusive breastfeeding at 6 months were available for 30 countries with a median of 18%; among the European countries (*n* = 16), this parameter varied between 39% (Netherlands and Spain) and 0.8% (Greece) [36]. Any breastfeeding at 6 months was reported for 20 countries with a median of 45%; among the European countries (*n* = 17), the numbers varied between 78% (Norway) and 4% (Northern Ireland) [36]. Data for continued breastfeeding at around 12 months were reported for 25 countries with a median of 29%; European countries (*n* = 14) showed values between 62% (Finland, 9 to 11 months) and 0% (Switzerland, >10 to 12 months) [36].

The above-listed indicators clearly demonstrate that the WHO recommendations for breastfeeding are only partially adhered to in Europe. Consequently, those infants who are partially breastfed will all receive some amounts of infant formula, whereas those who entirely fail to receive breastfeeding are dependent on infant formula as their main source of nutrition. In spite of the clear practical importance of the issue, information on infant formula consumption are surprisingly scarce in peer-reviewed medical journals. (Here, it should be noted that the plentiful Internet data sources on the issue might be biased by various interests including commercial ones).

The determinants and dynamics of commercial infant formula consumption were recently outlined for 77 countries of the world, including 24 countries from Europe [37]. Between 2005 and 2019, total standard milk formula (formula products marketed for infants aged 0 to 6 months, although some for 0 to 12 months) retails increased by 54.5% to 10.8 kg/child in all countries, whereas the corresponding value was a 17.8% increase to 29 kg/child in Europe [37]. In 2019, per-child standard infant formula sales were 29 kg in high-income countries (*n* = 37), 15.6 kg in upper-middle-income countries (*n* = 25), and 3.6 kg in lower-middle-income countries (*n* = 15) [37].

The clear trend of increasing standard infant formula use with higher income was corroborated by correlation analyses between various parameters of infant feeding and wealth in low- and middle-income countries (*n* = 87 to 90, depending on the parameter analyzed) [38]. The logarithm of the gross domestic product of the countries showed significant inverse correlations with exclusive breastfeeding under 6 months (r = −0.37, *p* < 0.001) and continued breastfeeding at 1 year (r = −0.74, *p* < 0.0001), as well as a significant positive correlation with infant formula consumption under 6 months (r = 0.70, *p* < 0.0001) [38]. Within-country analyses showed that continued breastfeeding at 1 year was significantly higher in children belonging to the poorest 20% of households compared to the wealthiest 20% in 40 countries (by around 30 percentage points on average) [38].

A recent analysis of national data from 126 countries found that standard infant formula sales (kg per child) are significantly inversely associated with national breastfeeding rates at 12 months (r = −070, *p* < 0.0001): for each additional kilogram of standard formula sold per child each year, breastfeeding was 1.9% lower (95% confidence intervals, 1.5% and 2.2%) [39].

The considerations outlined above clearly indicate that infant formula represents an unfortunately common nutritional source for healthy, full-term infants around the world and especially in Europe. Besides the primary goal of protecting, supporting, and promoting breastfeeding, the improvement of the nutritional composition of infant formula may serve as a secondary, additional goal in supporting growth and development in infancy.

Even the few data cited above clearly indicate that infant formula represents an unfortunately important nutritional source for healthy, full-term infants. Hence, research aimed to improve the nutritional composition of infant formula might be justified.

### 4.2. Effects of Docosahexaenoic Acid in Infant Formula

Both the first RCTs addressing the effect of DHA supplementation of formula on the fatty acid status of infants [40,41] and the first review articles on the topic [42,43] were published in the early 1990s. (In order to indicate our personal interest in and devotedness to the topic of the present review, here, we add that also one of us initiated an RCT [44] and co-authored a review [45] on DHA in infant formula some 30 years ago; 46 of the publications depicted on Figure 1 are related to us).

It is not an easy task to delineate which RCTs should be taken into consideration when potential effects of DHA supply to infant formula are evaluated. Several RCTs showed evidence of beneficial effects on visual function and in specific cognitive domains; however, early methodological approaches do not necessarily reflect current thinking, and this undermines the strength of evidence [46]. Fortunately, there is a Cochrane Database Systematic Review assessing whether supplementation of formula milk with LCPUFA (DHA plus arachidonic acid (C20:4n-6, AA) or DHA alone) is both safe and beneficial for full-term infants [47]. The authors identified 31 RCTs and included 15 of these on altogether 1889 infants in the review; trials reporting only biochemical outcomes were not eligible for inclusion. Among the 9 studies that investigated visual acuity, 4 studies reported beneficial effects, whereas the remaining 5 did not. Meta-analysis of three RCTs showed a significant benefit for sweep visual evoked potential acuity at the age of 12 months [47].

Among the 11 studies that assessed neurodevelopmental outcomes, 4 reported beneficial effects, whereas the remaining 7 did not. Two out of the nine studies that used the Bayley Scales of Infant Development reported beneficial effects; however, meta-analyses revealed no significant differences between n-3 LCPUFA and placebo groups at the age of 18 months. Better novelty preference measured by the Fagan Infant Test at 9 months and better problem solving at 10 months were reported in one–one studies [47]. Among the 13 studies that measured physical growth, no beneficial or harmful effects of supplementation were reported. Meta-analysis of five RCTs showed that the supplemented group had lower weight, but not height or head circumference, at the age of 12 months, whereas no difference was seen at the age of 18 months [47]. However, Grading of Recommendations Assessment, Development and Evaluation (GRADE) analysis of the outcomes indicated that the quality of evidence was low. The authors of the Cochrane review concluded that “Most of the included RCTs reported no beneficial effects or harms…” and “Routine supplementation of full-term infant milk formula with LCPUFA cannot be recommended this time” [47].

Opinions expressed in Cochrane reviews are usually attributed decisive importance, so there appears to be some controversy between the Cochrane opinion and the existing European recommendation of mandatory supplementation of infant formula with DHA [5]. This controversy may be at least partially explained by the extreme complexity of the question to be addressed in RCTs. Genetic factors including the gender of the infant, environmental factors including maternal diet during pregnancy, different dosages and forms of DHA supplementation, as well as widely different methods to assess various outcome parameters may all contribute to mudding the water of research on the developmental effects of supplementing infant formula with LCPUFA (Table 2).

The influence of the fatty acid desaturase (FADS) genotype on maternal and child polyunsaturated fatty acid (PUFA) and LCPUFA status has been addressed in a recent systematic review of 45, mostly observational studies [48]. Eight articles investigated the relationship of FADS genotype to PUFA status during pregnancy; all these studies reported an increased contribution of LA and ALA in minor allele carriers, mostly together with decreased product substrate ratios, indicative of the decreased functionality of FADS. Maternal genotype with the minor allele for FADS was associated with decreased cord blood DHA concentrations and with reduced DHA synthesis capacity shown by lower EPA-to-ALA ratios [48].

Sex-specific differences in EFA and LCPUFA statuses were also described [49]; a systematic review of 51 publications showed a significantly lower contribution of AA and DHA to plasma total lipids and phospholipids in men than in women [50]. This finding is in line with sex-specific differences in some outcomes in DHA supplementation studies, e.g., maternal DHA supplementation resulting in significantly better problem solving in girls, but not in boys, in parallel with significantly less vocabulary comprehension in boys, but not in girls [51]. These significant differences between girls and boys in their developmental response to the same supplementation of infant formula with DHA raise the question of whether balanced randomization according to sex is enough to exclude this bias or power calculation of the RCTs should be made separately for girls and boys.

Different maternal DHA status during pregnancy may result in different DHA status at the initiation of DHA supplementation to the infant. Indeed, in a systematic review of the FA composition of venous cord blood phospholipids in 13 different European countries, the contribution of DHA ranged between 3.6% and 8.6%, i.e., there were more than two-fold differences in DHA status at birth [52]. It may be assumed with good reason that similar DHA supplementation on different baseline DHA status might cause different developmental effects. On the other hand, the nutrient composition of the formula may play different roles in socioeconomic environments differently influencing the development of the infant.

The dosage of DHA supplementation to the infant formula might play a decisive role in the detectability of developmental effects. When different dosages of DHA supplementation (0.32%, 0.64%, and 0.96% of total FAs) were investigated within the same study, various developmental tests showed significant dose-dependent differences among supplementation groups [53]. To complicate matters, in one test, infants who received 0.64% and 0.96% DHA performed significantly better than the controls, whereas in another test, significant differences from controls were seen with 0.32% and 0.64% DHA, but not with 0.96% DHA [53]. Moreover, the source of DHA may also influence the efficacy of supplementation. In the 15 studies included in the Cochrane review discussed above [47], egg yolk phospholipids or triacylglycerols, various fish oils, evening primrose oil, as well as single cell oils were used, raising the question of the potentially different bioavailability of the same dose of DHA from the different sources. Furthermore, infant formula represents a complex food matrix in that the different presence of other FAs (e.g., EPA) or lipid-soluble antioxidants (e.g., alpha-tocopherol, beta-carotene) may also influence the efficacy of DHA supplementation.

Several reviews addressed the question of the optimal methodology of assessing cognitive and visual functions in infancy in general [54,55] and in connection with clinical trials in infants in particular [56,57], respectively. However, there appears to be no clear recommendation on choosing the methods for outcome assessment. There, it is small wonder that, in the Cochrane review discussed above [47], the 9 studies on visual acuity used 3 different methods (steady state and/or sweep visual evoked potentials, and/or Teller cards), and 1 study addressing neurodevelopment used at least 4 different methods (Bayley Scales of Infant Development Mental Developmental Index and/or Psychomotor Developmental Index, Fagan Infant Test, Brunet and Lezine test). Moreover, the timing of assessment was far from being standardized: e.g., sweep visual evoked potentials were assessed at the ages of 4, 6, 7 to 8, and 12 months. While numerous studies have found positive correlations between blood DHA levels and improvements in cognitive or visual function outcomes in infants, the results of RCTs have been mixed, likely due to study design heterogeneity [58].

In summary, the long list of potentially significant confounding variables in RCTs on DHA supplementation in infancy (Table 2) makes it understandable that no high-grade evidence was presented up to now and makes it somewhat unlikely that such evidence will be revealed in the immediate future.

## 5. Current Considerations with Docosahexaenoic Acid in Infant Formula

Regulatory inclusion of DHA into the FA composition of infant formula in Europe was preceded by three scientific opinions of the European Food Safety Authority (EFSA) [59,60,61]. In its first opinion, the EFSA considered DHA as a conditionally essential FA for infants, and an adequate intake of 100 mg per day was set for 7- to 24-month-old infants [59]. In its second opinion, the EFSA took also into account observed intakes of DHA from HM and considered an intake of 100 mg per day DHA adequate for the majority of infants aged 0 to 6 months as well [60]. In its third opinion, the EFSA discussed in great detail available data on the effect of addition of DHA to infant formula on various health outcomes and considered that DHA should be added to infant formula, even though there was no conclusive evidence for any health outcome effect beyond infancy [61].

When summarizing the reasons for proposing the addition of DHA to infant formula, the EFSA opinion emphasized, besides the traditional considerations of (a) the structural role of DHA in the nervous tissues and in the retina, (b) the accumulation of DHA in the developing brain, and (c) erythrocyte DHA status closer to those of the breastfed infants with DHA supplementation than with ALA supplementation alone, also (d) the lack of adequate RCTs to demonstrate that the purported biologically plausible effects should be taken into consideration [61].

Using the lack of high-quality studies as supporting information for a dietary decision might sound strange, or even false, from the aspect of evidence-based healthcare. However, evidence-based thinking should always consider not only the importance of evidence, but the practical limitations of generating evidence as well. The all-too-common conclusion that “further studies are needed” should sometimes be confronted with the skeptical question of whether reasonable further efforts will yield decisive information for drawing firm conclusions in the foreseeable future. In the field of research on the role of DHA in infant formula, both the track records (Figure 1) and the hurdles of further investigations (Table 2) indicate that available evidence may not be significantly surpassed anytime soon and can probably be used now to support the relatively minor dietary recommendation of the inclusion of DHA into infant formula. The lack of any evidence of harms [47] may further support the recommendation. The recommendation of mandatory inclusion of DHA in infant formula in Europe is in the focus of current scientific attention mostly due to the lack of a clear opinion on including also the biologically most-important n-6 LPUFA, AA, in the formula: as the regulation formulates: “Other long-chain (20 and 22 carbon atoms) polyunsaturated fatty acids may be added” [5]. Because also AA appears to be required for optimal neurodevelopment [62], it is a question under current debate whether formula for full-term infants should provide AA along with DHA [63,64]. Mandatory inclusion of DHA in infant formulas opened up the question of the optimal intake levels of the classic n-6 EFA, linoleic acid (C18:2n-6), in infant formula as well [65]. However, studies on fat essentiality established that AA alone is more efficacious than linoleic acid for preventing the clinical symptoms of EFA deficiency [66]. AA has very different biological functions compared to DHA, and the overwhelming majority of trials include both DHA and AA and test development specific to DHA [67]. The maintenance of a proper balance between DHA and AA in formula for term infants is an already set goal [68], but the optimal composition of the supplement still needs to be determined [69]. The information collected in the present review might be considered also in this decision-making.

## Figures and Tables

**Table 1:** Human milk (HM) intake, fat content in HM, contribution of docosahexaenoic acid (DHA) to the fatty acid (FA) composition of HM, and calculated DHA intake in prospectively followed, healthy, full-term infants.

	Age (Number of Infants)		
	1 Month (*n* = 126)	2 Months (*n* = 117)	3 Months (*n* = 108)
HM intake (g/day)	625 (135)	700 (169)	711 (166)
Fat content in HM (g/100 mL)	3.20 (1.27)	3.16 (1.18)	2.92 (1.23)
DHA in FA composition (%)	0.25 (0.11)	0.24 (0.11)	0.26 (0.09)
DHA intake (mg/day)	48.5 (25.5)	51.3 (20.2)	50.3 (17.1)

Values are the mean (standard deviation). Modified from Grote et al. [18].

**Table 2:** Confounding factors in randomized controlled trials on developmental effects of supplementing formula for full-term infants with docosahexaenoic acid (DHA).

Category of Confounding Factor	Recognized Biases
Genetic	Fatty acid desaturase genotype polymorphism
Environmental	Differences between boys and girls Maternal DHA status during pregnancy Socioeconomic status of the family
Dietary	Time duration of the supplementation Dosage of DHA Origin of DHA Fatty acid matrix of the formula Other nutritional matrix of the formula
Methodological	Timing of assessment Different growth measurements among studies Different assessment of visual acuity among studies Different assessment of neurodevelopment among studies

For references, see the related text.

## Data Availability

The data presented in this review are available upon request to the corresponding author.
